# The 14-3-3 Proteins as Important Allosteric Regulators of Protein Kinases

**DOI:** 10.3390/ijms21228824

**Published:** 2020-11-21

**Authors:** Veronika Obsilova, Tomas Obsil

**Affiliations:** 1 Department of Structural Biology of Signaling Proteins, Division BIOCEV, Institute of Physiology of the Czech Academy of Sciences, 25250 Vestec, Czech Republic; 2 Department of Physical and Macromolecular Chemistry, Faculty of Science, Charles University, 12843 Prague, Czech Republic

**Keywords:** 14-3-3, kinase, phosphorylation, RAF kinase, ASK1, CaMKK2, PI4KB, LRRK2, PKC

## Abstract

Phosphorylation by kinases governs many key cellular and extracellular processes, such as transcription, cell cycle progression, differentiation, secretion and apoptosis. Unsurprisingly, tight and precise kinase regulation is a prerequisite for normal cell functioning, whereas kinase dysregulation often leads to disease. Moreover, the functions of many kinases are regulated through protein–protein interactions, which in turn are mediated by phosphorylated motifs and often involve associations with the scaffolding and chaperon protein 14-3-3. Therefore, the aim of this review article is to provide an overview of the state of the art on 14-3-3-mediated kinase regulation, focusing on the most recent mechanistic insights into these important protein–protein interactions and discussing in detail both their structural aspects and functional consequences.

## 1. Introduction

Phosphorylation by kinases governs many key cellular and extracellular processes, such as transcription, cell cycle progression, differentiation, secretion and apoptosis. Tight and precise kinase regulation is a prerequisite for normal cell functioning. Conversely, kinase dysregulation often leads to disease. The function of many kinases is regulated through protein–protein interactions. These interactions are in turn mediated by phosphorylated motifs and often involve associations with the scaffolding and chaperon protein 14-3-3.

14-3-3-mediated regulation has already been demonstrated for more than 30 kinases, and more than 170 kinases contain phosphosites known to conform to 14-3-3-binding sites [1]. However, many details concerning these interactions, especially the exact role of 14-3-3 binding and the mechanism of action of 14-3-3-mediated kinase regulation, remain elusive because only a few 14-3-3:kinase complexes have been structurally characterized so far, mostly using low-resolution approaches [2,3,4]. Nevertheless, several structures of rapidly accelerated fibrosarcoma B-type (B-RAF) kinase bound to 14-3-3 have been recently solved, thus providing the first glimpse, at atomic resolution, into a structural mechanism of 14-3-3-mediated kinase regulation [5,6,7,8].

Considering the above, the aim of this review article is to provide an overview of the current knowledge on 14-3-3-mediated kinase regulation. Although previous studies have shown that 14-3-3 proteins are involved in the regulation of many kinases, this review focuses only on complexes, whose available data enabled more detailed mechanistic insight into the 14-3-3-mediated regulation. Based on these data, we discuss structural aspects and functional consequences of these important protein–protein interactions.

## 2. 14-3-3 Proteins

**Figure 1:**
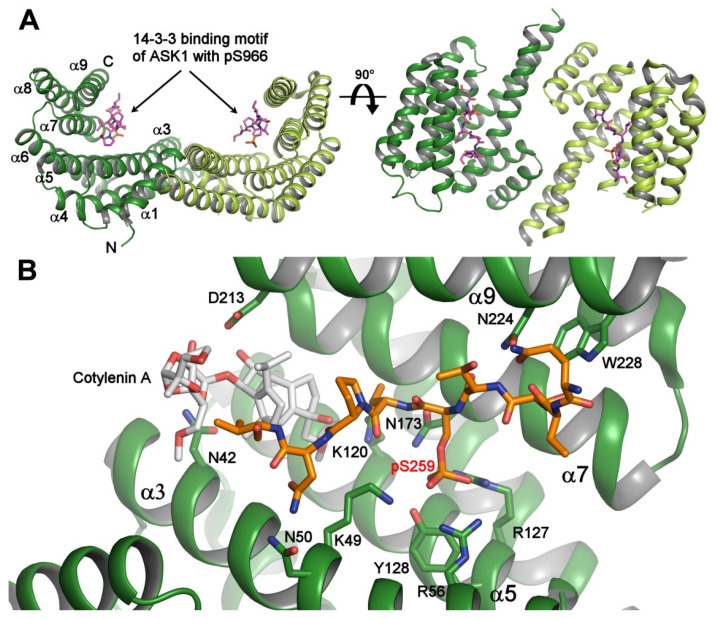
Crystal structure of the 14-3-3 protein with bound phosphopeptides. (**A**) The 14-3-3 binding motif of apoptosis signal-regulating kinase 1 (ASK1) (sequence RSIpS^966^LPVP) bound to human 14-3-3ζ (PDB ID: 6EJL). The 14-3-3 protein molecule is a dimer with a two-fold symmetry, and each protomer consists of nine antiparallel α-helices and contains an amphipathic groove which is a binding site for the phosphorylated motifs; (**B**) the ternary complex between human 14-3-3σ (shown in green), the 14-3-3 binding motif pSer^259^ of C-RAF (shown in orange), and Cotylenin A (shown in gray) (PDB ID: 4IHL [21]). The 14-3-3σ residues that make polar contacts with the phosphopeptide and Cotylenin are shown as sticks. The Cotylenin A considerably enhances the binding of the C-RAF pSer^259^-motif to 14-3-3. The figure was prepared with PyMOL (https://pymol.org/2/).

The 14-3-3 protein family encompasses structurally similar acidic proteins found in all eukaryotes. Although 14-3-3 proteins were originally characterized as abundant brain proteins in 1967, with no clear function, they were later identified as universal adaptor and chaperon proteins, known for interacting with hundreds of other proteins and for participating in the regulation of almost every major cellular pathway [9,10,11,12]. Since their discovery, seven human 14-3-3 isoforms have been identified α/β, γ, ε, ζ/δ, η, τ, σ, where α and δ are the phosphorylated forms of the β and ζ isoforms [13]. Other organisms, such as budding yeast, contain only two isoforms, Bmh1 and Bmh2, whereas plants express 15 different 14-3-3 isoforms [14,15]. In addition, 14-3-3 proteins form a stable homo- or heterodimers, with each protomer consisting of nine tightly packed antiparallel α-helices, which create an amphipathic ligand-binding groove (Figure 1A) [16,17]. The 14-3-3 dimers can be destabilized by the lipid sphingosine and phosphorylation of Ser^58^ at the dimer interface [18,19] and it has been shown that such destabilization induces downregulation of Raf-MAPK and PI3K-Akt signaling [20].

## 3. 14-3-3 Protein-Dependent Regulation of Selected Kinases

Phosphorylation by protein kinases governs many key cellular and extracellular processes by participating in various signaling pathways. Therefore, tight kinase regulation is a key prerequisite for normal cell functioning and its dysfunction often leads to disease. Many kinases are regulated in the 14-3-3 protein-dependent manner; however, the structural data available so far enable a detailed understanding of these regulations for only a few kinases. In this review article, we discuss regulation of RAF, ASK1, leucine-rich repeat protein kinase-2 (LRRK2), protein kinase C (PKC), calcium/calmodulin-dependent protein kinase kinase (CaMKK), and phosphatidylinositol-4-kinase-IIIβ (PI4KB), for which the mechanistic insights into their 14-3-3 protein-mediated regulation are available.

### 3.1. RAF Kinases

RAF kinases are key components of the RAS-RAF-MEK-ERK signaling pathway. These kinases were among the first oncoproteins characterized in the 1980s, and B-RAF is the most frequently mutated protein kinase in various cancers, with V600E standing out as the most common and resistant mutation [22,23]. Selective inhibitors targeting RAF proteins are currently at various stages of clinical trials because RAF activity is strictly regulated, and any misbalance in ERK signaling activation or dysregulation usually leads to cancer.

**Figure 2:**
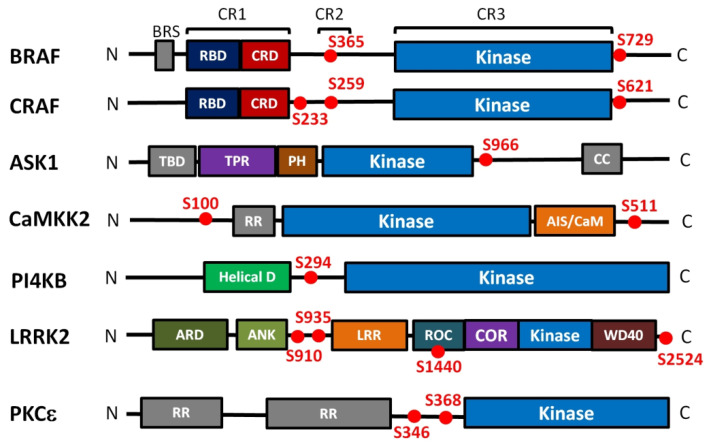
Domain structure and positions of 14-3-3 binding motifs of kinases regulated in the 14-3-3-dependent manner. Kinase domains are shown in blue, the 14-3-3 binding sites are shown as red dots. BRS, B-RAF specific domain; RBD, Ras binding domain; CRD, cysteine rich domain; TBD, TRX-binding domain; TPR, tetratricopeptide repeats domain; PH, pleckstrin homology domain; CC, coiled-coil region; RR, N-terminal regulatory site; AIS/CaM, autoinhibitory segment/CaM-binding domain; Helical, helical domain; ARD, armadillo repeat; ANK, ankyrin repeat; LRR, leucine-rich repeat; ROC, Ras of the complex GTPase domain; COR, C-terminal of ROC; WD40, WD40 or beta-transducin repeat. The kinases differ in their length and they are not shown on the same scale.

Mammalian RAF kinases (A-RAF, B-RAF and C-RAF) share the same domain organization, consisting of three conserved regions (CR1-CR3) (Figure 2). The CR1 region contains the RAS-binding domain (RBD) and the cysteine-rich domain (CRD), the CR2 region is rich in Ser/Thr and contains an inhibitory 14-3-3 binding site (Ser^259^ in C-RAF, Ser^365^ in B-RAF), and the third CR3 region comprises the kinase domain and a dominant activatory 14-3-3 binding site (Ser^621^ and Ser^729^ in C-RAF and B-RAF, respectively). C-RAF contains an additional inhibitory 14-3-3 binding site Ser^233^ located between CR1 and CR2 [24]. In the autoinhibited state, the N-terminal region of RAF interacts with the catalytic domain, thus inhibiting its kinase activity [22,25]. Furthermore, the inactive state of RAF is maintained by the 14-3-3 protein dimer, which simultaneously recognizes both phosphorylated motifs bordering the catalytic domain [6,7,24,26].

Mitogen stimulation induces RAS-GTP binding to the RAF RBD, which anchors RAF to the plasma membrane, followed by dephosphorylation of the 14-3-3 binding sites within the CR2 by PP1 and PP2A phosphatases. This dephosphorylation releases the 14-3-3 protein from this site and enables RAF binding to the plasma membrane through the CRD. As a result, RAF moves closer in proximity to other kinases involved in this process (SRC family kinases and casein kinase 2), which phosphorylate several activating sites within the N-terminal region of RAF.

The key event in RAF activation is the 14-3-3-mediated dimerization of the RAF kinase domain, which promotes the catalytic activity of RAF. RAF dimerization is stabilized by the 14-3-3 dimer, which simultaneously binds two RAF molecules through its C-terminal 14-3-3 binding motifs located within the CR3 C-terminal to the kinase domain [5,6,7,8,27]. Activated RAF then phosphorylates MEK, which activates ERK, which, in turn, activates a wide range of targets associated with growth, proliferation, differentiation, survival, and migration [22]. Negative feedback is facilitated by ERK phosphorylation of several distinct inhibitory sites within the N-terminal domains of RAF followed by RAF dissociation from the activated RAS and disruption of RAF dimers.

14-3-3 proteins were identified as key RAF-binding partners, which control RAF activity, in 1994 [28,29,30,31]. The crystal structure of the peptide containing the motif around phosphoserine pSer^259^ from the CR2 of C-RAF bound to 14-3-3 was one of the first structures of the 14-3-3:phosphopeptide complexes ever published, showing how 14-3-3 proteins recognize phosphorylated targets [32]. Three 14-3-3 binding sites were identified in C-RAF: inhibitory Ser^233^ between CR1 and CR2, inhibitory Ser^259^ within CR2 and activatory Ser^621^ in CR3 [24,26]. C-RAF phosphorylation by PKA promotes its association with the 14-3-3 protein via Ser^233^ and Ser^259^ [24]. The crystal structures reported by the Ottmann group indicated that the 14-3-3 dimer can simultaneously recognize both phosphorylated motifs Ser^233^ and Ser^259^, with pSer^259^ functioning as a high affinity binding site and pSer^233^ as a low-affinity binding site [33]. Moreover, both inhibitory 14-3-3 binding sites N-terminal to the kinase domain (Ser^233^ and Ser^259^), but not the activatory site Ser^621^, can be stabilized by small molecule compounds targeting cavities at the 14-3-3:C-RAF interface (Figure 1B) [21,34]. Accordingly, such stabilization of the interaction between 14-3-3 and the inhibitory C-RAF 14-3-3 binding motifs may be an effective strategy for the treatment of RAS-connected diseases [21].

**Figure 3:**
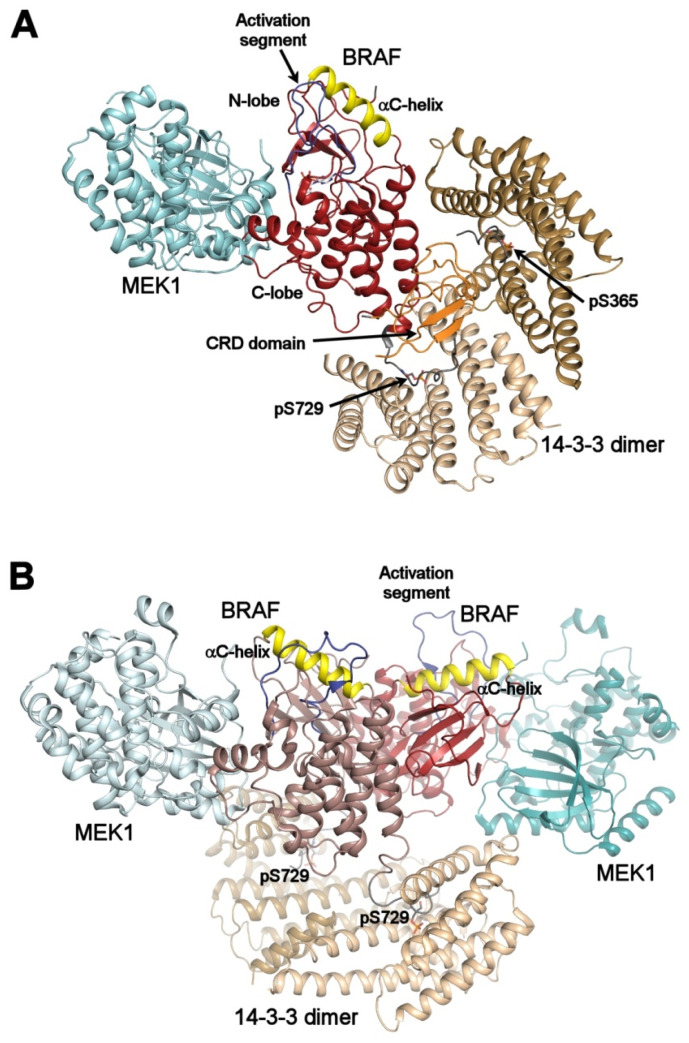
Autoinhibited and active B-RAF:MEK1:14-3-3 complexes. (**A**) structure of the autoinhibited BRAF:MEK1:14-3-3 complex (PDB ID: 6NYB [6,7,24,26]). The 14-3-3 dimer simultaneously binds both pSer^365^ and pSer^729^ motifs (shown in gray) bordering the B-RAF kinase domain (shown in dark red). The CRD domain (shown in orange) is sequestered within the central channel of the 14-3-3 dimer. The position of the αC-helix (shown in yellow) and the activation segment (shown in blue) correspond to the autoinhibited state. The 14-3-3 protein inhibits B-RAF by blocking its membrane localization dimerization through steric occlusion; (**B**) structure of the active B-RAF:MEK1:14-3-3 complex (PDB ID: 6Q0J [6,7,24,26]). Dephosphorylation of the N-terminal motif (pSer^365^) causes structural rearrangement resulting in an active B-RAF dimer stabilized by the 14-3-3 dimer through anchoring C-terminal pSer^729^ motifs of two B-RAF molecules. The B-RAF kinase domains are oriented in the back-to-back fashion with the αC-helix in a position consistent with the active conformation. The figure was prepared with PyMOL (https://pymol.org/2/).

The crucial step in both normal and disease-connected RAF signaling is RAF dimerization through its kinase domains, and any destabilization of RAF dimers formation may contribute to RAF inhibition [35,36]. The recently reported cryo-EM and crystal structures of 14-3-3:B-RAF complexes have provided detailed mechanistic insights into how 14-3-3 proteins regulate B-RAF activity both negatively and positively (Figure 3) [5,6,7,8].

Negative regulation: In the structure of the autoinhibited B-RAF:MEK1:14-3-3 complex, the 14-3-3 protein simultaneously binds phosphorylated Ser^365^ and Ser^729^ motifs bordering the kinase domain of one B-RAF molecule sequesters the CRD of B-RAF within the central channel of the 14-3-3 dimer and obstructs the dimerization interface of the B-RAF kinase domain (Figure 3A). Both the position of the C-helix and the conformation of the activation segment in the kinase domain correspond to the autoinhibited state. Thus, the 14-3-3 protein appears to keep B-RAF in an inactive state by blocking the membrane recruitment of B-RAF and by preventing B-RAF kinase dimerization through steric occlusion.

Positive regulation: In turn, the structure of the active dimeric B-RAF:MEK:14-3-3 complex revealed that B-RAF dephosphorylation at Ser^365^ induces a structural rearrangement, resulting in an active B-RAF dimer stabilized by the 14-3-3 dimer, which anchors Ser^729^-containing C-terminal segments of two B-RAF molecules (Figure 3B). Within this complex, the B-RAF kinase domains are oriented in a back-to-back fashion, with the C-helix in an inward position, in line with the active conformation.

### 3.2. Apoptosis Signal-Regulating Kinase 1 (ASK1)

ASK1 belongs to a group of mitogen-activated protein kinase kinase kinases (MAP3Ks). The hierarchical system of the mitogen-activated protein (MAP) kinase cascade is formed by upstream kinases MAP3Ks that phosphorylate intermediate MAP kinases MAP2Ks (MKK3, MKK4 and MKK6) which in turn phosphorylate terminal MAP kinases (p38, JNK or ERK) [37]. Active, phosphorylated MAPKs phosphorylate a wide range of substrates to stimulate cell proliferation, cell death, or inflammation [38]. The aberrantly enhanced ASK1-MAPK signaling leads to many disorders and neurological diseases, such as amyotrophic lateral sclerosis, multiple sclerosis and Parkinson’s, Alzheimer´s, and Huntington´s disease. Due to the complexity of the signaling processes in which ASK1 participates, mechanistic and structural insights into the ASK1 signaling pathways are necessary for developing inhibitors as therapeutical targets against these devastating diseases [39].

Human ASK1 contains a thioredoxin binding domain (TBD) at the N-terminus, the central regulatory region (CRR) with a tumor necrosis factor receptor associated factor (TRAF)-binding region, a catalytic domain (CD), in the middle of the molecule, and C-terminal coiled-coiled (CC) and sterile-alpha motif (SAM) domains, which are involved in ASK1 homo- and hetero-oligomerization with the closely related ASK2 [40,41,42,43].

Under normal, non-stress conditions, ASK1/ASK2 hetero-oligomers interact with several other proteins, including thioredoxin (TRX) and 14-3-3, forming a high molecular mass complex known as the ASK1 signalosome [44]. Federspiel et al. have shown that the ASK1 signalosome contains ASK2 at a 1:1 stoichiometric ratio with ASK1 and 14-3-3 proteins [45]. The 14-3-3 protein binds to the phosphorylated motifs containing pSer^966^ and pSer^964^ in ASK1 and ASK2, respectively, located C-terminally to the CD domains, suppressing both the autokinase and the transkinase activities of ASK1 (Figure 2) [46,47]. Both 14-3-3 and TRX are physiological inhibitors of ASK1.

Under stress conditions, 14-3-3 and TRX dissociate from ASK1. Then, the N-terminal parts of ASK1/ASK2 homo-oligomerize, and TRAF 2 and 6 bind to the CRR domain(s). After autophosphorylation of ASK1 Thr^838^ within the activation loop, ASK1 is activated, phosphorylating its downstream targets [46,48,49,50]. The crystal structure of the ASK1-CRR domain showed that a conserved surface of CRR, formed by the pleckstrin homology (PH) domain and the tetratricopeptide repeat (TPR) region, is necessary for ASK1 activity. CRR has a dual role: on the one hand, it stimulates ASK1 activity through the PH domain; on the other hand, it aids ASK1 autoinhibition by delivering the TBD and CD domains near each other [51].

The closeness of the 14-3-3 binding motif (Ser^966^) to ASK1-CD suggests that the 14-3-3 protein modulates the enzyme activity or accessibility of its active site, as previously shown for other enzymes regulated by 14-3-3 [52,53]. The low-resolution structural characterization of the complex between ASK1-CD and 14-3-3 indicated that the 14-3-3 protein might inhibit ASK1 by modulating the structure of its active site, by sterically blocking the phosphorylation of Thr^838^ within the activation loop and by blocking the interactions between ASK1 and its substrates [2]. Moreover, ASK1-CD forms a stable dimer in solution, and its complex with the 14-3-3 protein, which is also dimeric, is conformationally heterogeneous, lacking a clearly defined interface, presumably due to the absence of another protein, such as ASK2 [46,54]. The SAXS-based structural model of the ASK1:14-3-3ζ complex also suggest that the pSer^966^ in one protomer of the ASK1 dimer binds to one protomer of the 14-3-3 dimer, leaving the other protomers of 14-3-3 and ASK1 ligand-free [2].

Because the 14-3-3 protein is a physiological inhibitor of ASK1, the ASK1:14-3-3 and ASK2:14-3-3 complexes are potential targets for therapeutic intervention as an alternative or complimentary strategy in suppressing the ASK1 activity. Recent advances in the development of small molecule compounds aimed at stabilizing 14-3-3 protein-kinase interactions [21,55,56,57,58] have demonstrated the feasibility of this approach.

### 3.3. Calcium/Calmodulin-Dependent Protein Kinase Kinases (CaMKK)

CaMKKs are Ser/Thr protein kinases that function as upstream elements of the CaMK signaling cascade where they phosphorylate and activate two downstream CaMKs, CaMKI and CaMKIV [59,60,61,62]. Upstream extracellular signals, including insulin, hormones (insulin, thyroid hormone, growth hormone), glucose and amino acids, trigger the increase in the intracellular concentration of free Ca^2+^, which is followed by the activation of Ca^2+^/CaM targets such as CaMKs. In the inactive state, the kinase activity of CaMKs is blocked by the auto-inhibitory segment (AIS), which interacts with the kinase domain (KD) and prevents substrate binding and/or structurally modulates the catalytic site [63]. This autoinhibition is relieved by Ca^2+^/CaM binding to the region overlapping with the AIS. CaMKI participates in the regulation of hippocampal memory formation, neuronal migration, cell survival and synaptogenesis, whereas CaMKIV is involved in the control of protein synthesis and gene expression programs in response to nutrients and hormones [64,65].

Mammalian CaMKKs include two isoforms: CaMKK1 and CaMKK2. These isoforms have a similar domain organization, that is, the catalytic kinase domain is followed by the C-terminal AIS, which partly overlaps with the Ca^2+^/CaM-binding region [62,66]. However, these CaMKK isoforms exhibit different biochemical properties because CaMKK1 is strictly regulated in a Ca^2+^/CaM-dependent manner, whereas CaMKK2 also shows considerable Ca^2+^/CaM-independent activity. In addition, CaMKK2 is an important upstream activator of the histone deacetylase SIRT 1 and AMP-activated protein kinase (AMPK) [67,68], a key regulator of energy homeostasis, inflammation, and autophagy whose dysregulation has been implicated in chronic diseases such as obesity, diabetes and cancer [60,69].

Both CaMKK isoforms are partly inhibited upon phosphorylation by PKA at multiple sites in a process involving 14-3-3 protein binding. CaMKK1 possesses five PKA phosphorylation sites (Ser^52^, Ser^74^, Thr^108^, Ser^458^, and Ser^475^), whereas CaMKK2 contains only four such sites (Ser^100^, Thr^145^, Ser^495^, and Ser^511^). Two sites phosphorylated by PKA are responsible for 14-3-3 protein binding; the first site is located at the N-terminus (Ser^74^ in CaMKK1; Ser^100^ in CaMKK2), and the second site is on the C-terminus (Ser^475^ in CaMKK1; Ser^511^ in CaMKK2) (Figure 2) [70,71,72,73]. 14-3-3 protein recruitment by CaMKK1 partly suppresses its catalytic activity, whereas CaMKK2 activity remains unaltered, suggesting different regulation mechanisms for these isoforms [3,70,71,74]. Moreover, it has recently been shown that phosphorylation of CaMKK2 at Ser^495^ is involved in regulating VEGF-induced AMPK activation, a pathway shown to regulate angiogenesis [75].

Low-resolution structural analysis of the CaMKK2:14-3-3 complex indicated that the formation of the complex affects the structure of several regions of CaMKK2 outside the 14-3-3 binding motifs, including the regulatory region within the N-terminal extension and the C-lobe of the kinase domain, which are apparently located outside the central channel of the 14-3-3γ dimer [3]. Nevertheless, previous studies on both CaMKK isoforms suggested that the main role of 14-3-3 protein binding in CaMKKs regulation is to slow down their dephosphorylation, especially at the serine residue located within the Ca^2+^/CaM-binding region, whose phosphorylation has been shown to block Ca^2+^/CaM binding. This, in turn, keeps CaMKKs in their PKA-mediated inhibited states [3,70,74].

Furthermore, the crystal structure of the N-terminal 14-3-3 binding motif of CaMKK2 (Ser^100^) bound to 14-3-3 revealed that it adopts an unusual conformation within the 14-3-3 ligand binding groove (PDB ID: 6EWW) [3]. The N-terminal motif (sequence RKLpS^100^LQE) does not conform to any canonical 14-3-3 binding motif because it contains a Gln residue and not a Pro residue at position +2 relative to the phosphorylated residue pSer100 [3]. In this crystal structure, the asymmetric unit contained four copies of the phosphopeptide:14-3-3 complex and in three of them the side chain of Gln^102^ interacts with pSer^100^, likely forcing the direction of the polypeptide chain to change and thus mimicking the role of the Pro residue. Due to the abrupt change in the direction of the polypeptide chain, the part of the 14-3-3 ligand binding groove known as “the fusicoccin binding cavity” remains empty. As such, the interaction between CaMKK2 and 14-3-3 may be stabilized by small-molecule compounds, as previously described for other 14-3-3 complexes [34,57,58,76]. Indeed, the recently published study confirmed that fusicoccins can stabilize protein–protein interactions between CaMKK2 and 14-3-3 [56]. On the other hand, structure of the diphosphorylated pSer^100^-pSer^511^ peptide (PDB ID: 6EF5) did not show the interaction between Gln^102^ and pSer^100^, thus suggesting that the proline-like conformation of the polypeptide chain can also be formed without that interaction [74].

### 3.4. Phosphatidylinositol 4-Kinases

Phosphoinositides (PIPs) are phosphorylated derivatives of phosphatidylinositol (PI). Seven PIPs have been described in eukaryotes with the combination of 1-3 phosphates in positions 3, 4, and 5 of the inositol ring. PIPs are critically involved in numerous cellular processes, such as signal transduction and vesicular trafficking, modulating lipid distribution and metabolism, regulating ion channels, pumps, and transporters and controlling both endocytic and exocytic processes [77,78]. Among the PIPs, phosphatidylinositol 4,5-bisphosphate (PI(4,5)P_2_) stands out as the most abundant anionic lipid. Its synthesis is primarily mediated by the PIP5K group of phosphatidylinositol kinases, which phosphorylate phosphatidylinositol 4-phosphate (PI(4)P) at position 5 of the inositol ring. PI(4,5)P_2_ phosphorylation by PI3K yields PI(3,4,5)P_3_, which is a potent signal for survival and proliferation, whereas PI(4,5)P_2_ hydrolysis by phospholipase C yields two second messengers: inositol trisphosphate (cytosolic) and diacylglycerol (membrane-bound). Together, these second messengers initiate several downstream signaling cascades [79].

Phosphoinositide 4-kinases (PI4K), which synthesize PI(4)P, are classified into two groups: type II and type III (the missing type I kinase is a historical artefact). Type II kinases are not similar to any other lipid kinase. In contrast, type III kinases are typical PI4Ks because they resemble PI3Ks. Type III phosphatidylinositol-4-kinases are hijacked by various +RNA viruses, such as viruses from the *Picornaviridae*, *Flaviviridae*, and *Coronaviridae* families. For this reason, these enzymes are attractive targets for pharmaceutical intervention [80,81].

Phosphatidylinositol-4-kinase-IIIβ (PI4KB) is a class III PI4K kinase with a domain organization very similar to PI3K. This kinase has an N-terminal helical domain and a C-terminal catalytic domain. The catalytic domain consists of N- and C-lobes, with an internal linker in the N-lobe [78]. Several binding partners of PI4KB have been identified, including the adaptor protein Acyl-CoA-binding protein 3 (ACBD3, often also abbreviated as ACBP) [82], the small GTPase Rab11 [83] and the 14-3-3 protein [84]. The ACBD3 interacts with the PI4KB region preceding the helical domain and is important for enterovirus replication via its interactions with viral proteins and PI4KB. Rab11 interacts with the helical domain of PI4KB, and 14-3-3 protein recognizes the phosphorylated motif around Ser^294^ located within the flexible linker that connects the helical domain to the catalytic domain (Figure 2) [4,85,86,87]. The 14-3-3 binding site of PI4KB is conserved across the mammalian and yeast species [88], and its phosphorylation is mediated by protein kinase D (PKD) [89]. The PKD-mediated phosphorylation of PI4KB is associated with the Golgi compartment and stimulates the lipid kinase activity of PI4KB [84,90]. However, this activation is not caused by the 14-3-3 protein, whose recruitment has no direct effect on PI4KB activity or on its nucleocytoplasmic shuttling, instead increasing the stability of PI4KB and protecting it against dephosphorylation [4,84]. In addition, 14-3-3 may also regulate PI4KB through 14-3-3 dimers, which recruit other 14-3-3 binding proteins [91].

The crystal structure of yeast 14-3-3 protein with a bound synthetic phosphopeptide derived from the human PI4KB lipid kinase revealed interactions typical of 14-3-3:phosphopeptide complexes [87]. Recently, biophysical characterization of the complex between full-length PI4KB and 14-3-3 has shown that the complex is tight and formed in a 2:2 molar stoichiometry. Low-resolution structural analysis by SAXS indicated that the complex is compact but flexible, especially within the disordered loops connecting the 14-3-3 binding site to the rest of the enzyme, thus allowing multiple orientations between PI4KB and the 14-3-3 protein dimer. The resulting models have also shown unrestricted access to the active site of PI4KB and a small binding interface between 14-3-3 and PI4KB [4].

Interestingly, SAXS-based analysis of the ternary 14-3-3:PI4KB:Rab11 complex highlighted a compact arrangement in a 2:1:1 stoichiometry, that is, one 14-3-3 dimer binds one molecule of phosphorylated PI4KB, which binds one molecule of Rab11 [92]. The orientation of PI4KB with respect to the 14-3-3 dimer is different from that of the binary 14-3-3:PI4KB complex, but the active site of PI4KB is also fully exposed. The disordered C-terminal tail of Rab11 adopts an extended conformation, with no direct contact with 14-3-3, which suggested that 14-3-3 does not interfere with Rab11 binding to the membrane via its C-terminus. The 14-3-3 dimer bound to PI4KB may also play an additional role, as suggested by Wortzel et al. [93], who showed that the ERK1c kinase phosphorylated at Ser^343^ interacts with the 14-3-3:PI4KB complex, thus enabling ERK1c translocation to the Golgi, where ERK1c is phosphorylated by MEK1b and induces mitotic Golgi fragmentation. Therefore, the 14-3-3 protein not only stabilizes PI4KB, protecting it against dephosphorylation, but also enables crosstalk between lipid signaling pathways and the ERK cascade.

### 3.5. Leucine-Rich Repeat Protein Kinase-2 (LRRK2)

LRRK2 is a large multidomain Ser/Thr protein kinase of the Roco family with a characteristic ROC (Ras of the complex)-COR (C-terminus of ROC) bi-domain [94]. These two domains interact with each other, but ROC has a GTPase activity, whereas COR functions as a dimerization module [95]. LRRK2 mutations, either in the kinase or in the Roco-type GTPase domains, are the most prevalent cause of familial Parkinson´s disease (PD) [96]. Therefore, developing new strategies for the treatment of PD treatment requires understanding the regulatory mechanism of this protein [97].

LRRK2 is phosphorylated on multiple sites in vivo, and the phosphorylation of two or more residues (Ser^910^, Ser^935^, Ser^1444^ in the ROC domain, and Thr^2524^ at the very C-terminus) triggers its association with the 14-3-3 protein. Among these phosphorylated residues, pSer^910^, pSer^935^, and pSer^1444^ account for the tightest binding (Figure 2) [98,99,100,101,102]. In addition, several PD-associated LRRK2 mutants suppress phosphorylation on these sites, thereby also reducing 14-3-3 protein binding. Accordingly, Muda et al. [101] demonstrated that disrupting the interaction between the LRRK2 ROC domain and 14-3-3 by introducing the R^1441^H and S^1444^A mutations increases LRRK2 kinase activity, whereas in vitro LRRK2 WT phosphorylation by PKA at Ser1444 in the presence of 14-3-3 decreases LRRK2 activity.

The autophosphorylation site Thr^2524^ located at the very C-terminus of LRRK2 in the WD40 domain has been recently identified as another 14-3-3 binding motif resembling the C-terminal canonical mode III [100]. Deletion of the C-terminal segment abolished both the kinase activity and 14-3-3 protein binding, thus highlighting the importance of the C-terminus for LRRK2 function, as corroborated by the recently reported cryo-EM structure of the LRRK2 construct with ROC-COR-kinase-WD40 domains. This structure revealed that the C-terminal segment is located near the N-lobe of the kinase domain and the adjacent COR domain [103].

Because the kinase activity of LRRK2 is enhanced in PD and reduced upon binding to 14-3-3, stabilizing the interaction between LRRK2 and 14-3-3 using small-molecule compounds is another potential therapeutic strategy for PD. The structure of the doubly phosphorylated LRRK2 peptide containing both pSer^910^ and pSer^935^ bound to 14-3-3 reported by the Ottmann group suggested the “drugability” of this interface, especially at the site Ser^935^, which contains a pocket targetable by compounds such as the fungal toxin Fusicoccin A [55,99].

Notwithstanding the advances described above, the role of 14-3-3 protein in regulating LRRK2 is far from being fully understood. More specifically, the mechanism whereby 14-3-3 protein suppresses LRRK2 kinase activity remains unclear and may involve interference with the dimerization of the ROC domain, modulation of the interaction between ROC and the adjacent COR domain or even interaction of the C-terminal segment with the kinase and COR domains [100,101,103]. Hopefully, the recently reported cryo-TM and cryo-EM structures of LRRK2 will soon be followed by structural studies aimed at clarifying the role of 14-3-3 in LRRK2 function [103,104].

### 3.6. Protein Kinase C (PKC)

The PKC family consists of evolutionary conserved calcium and phospholipid-dependent Ser/Thr protein kinases implicated in multiple signal transduction networks [105,106]. In total, nine mammalian PKC genes are divided into three subfamilies: conventional (PKC α, β and γ), novel (PKC δ, ε, θ, η) and atypical (PKC ζ and ι) PKCs. All PKCs share a common architecture of the N-terminal regulatory element connected by a flexible link to a C-terminal catalytic kinase domain [107]. Variable regions within the structure (V1–V5) define the isoform-binding specificity and enable the signal diversity of PKCs [108].

In particular, PKCε controls cytokinesis, the final stage of the cell cycle during which cells divide into daughter cells. This process involves the PKCε:14-3-3 interaction [109,110]. In mitotic cells, 14-3-3 proteins activate the phosphorylated PKCε in the absence of lipids. The selective inhibition of the 14-3-3:PKCε complex formation leads to defects in the completion of cytokinesis. This delay is connected with the prolonged localization of Ras Homolog Family Member A (RhoA) at the midbody, the enhanced RhoA activity, and the delay in actomyosin ring dissociation. In addition, incomplete cytokinesis can promote tumorigenesis [111]. Consequently, the regulatory mechanism of this process is still intensively studied.

Three upstream activators, glycogen synthase kinase 3 (GSK3), p38 MAP kinase and PKC itself, phosphorylate PKCε residues Ser^346^, Ser^350^, and Ser^368^, respectively [110]. All three sites are located in the V3 hinge region that connects the regulatory N-terminal part to the C-terminal kinase domain and conserved among vertebrates. Furthermore, motifs surrounding Ser^346^ and Ser^368^ were identified as 14-3-3 binding sites, and their phosphorylation induces the formation of the PKCε:14-3-3 complex in a 1:2 stoichiometry (Figure 2). The site containing Ser^346^ is a canonical mode I motif, whereas the second site somewhat resembles mode II motif for its lack of a Pro residue at +2 position with respect to pSer^368^.

The Ser^368^ site is a gatekeeper (dominant) site, as shown by structural analysis combined with calorimetry measurements. However, high affinity binding requires the presence of both phosphorylated motifs [112]. Interestingly, simultaneous phosphorylation of the third site, Ser^350^, substantially reduced the binding affinity of the phosphopeptide containing the V3 hinge region. Thus, the phosphorylation status of this site may function as an additional modulator of the 14-3-3-mediated activation of PKCε [112].

Functionally, 14-3-3 protein binding has been shown to activate PKCε in the absence of lipids [110]. This suggests that 14-3-3 stabilizes the active open conformation of PKCε and promotes its accumulation at the midbody by relieving or blocking an inactive conformation in which the active site of the kinase domain is occupied by the N-terminal pseudosubstrate region.

## 4. Conclusions and Challenges

In this review article, we demonstrated that 14-3-3 proteins are crucial regulators of many physiologically relevant kinases. However, the data available so far enable a detailed understanding of 14-3-3 protein-mediated regulations for only a few kinases. A *bona fide* exception is the B-RAF kinase. The recently solved high-resolution structures of B-RAF:14-3-3 complexes have shed light into the role of 14-3-3 binding. Accordingly, we hope that these structures will stimulate further structural studies of other 14-3-3:kinase complexes towards a more complete understanding of all nuances of these protein–protein interactions and their roles in regulating kinase functions. Concomitantly, 14-3-3 proteins are physiological “inhibitors” of several kinases whose dysregulation is involved in cancer and neurodegenerative diseases (e.g., RAF, ASK1, LRRK2). As such, these complexes are potential targets for therapeutic interventions. In fact, recent advances in the development of small molecule compounds aimed at stabilizing 14-3-3 protein–protein interactions have demonstrated the feasibility of this approach as an alternative or complimentary strategy in suppressing the activity of these kinases.

## Abbreviations

ACBD3	Acyl-CoA-binding protein 3
AMPK	AMP-activated protein kinase
ASK1	Apoptosis signal-regulating kinase 1
CaMKK	Calcium/calmodulin-dependent protein kinase kinase
GSK3	Glycogen synthase kinase 3
LRRK2	Leucine-rich repeat protein kinase-2
MAP	Mitogen-activated protein
MAPK	Mitogen-activated protein kinase
MAP2K	Mitogen-activated protein kinase kinase
MAP3K	Mitogen-activated protein kinase kinase kinase
PD	Parkinson’s disease
PI	Phosphatidylinositol
PIP	Phosphoinositides
PI4K	Phosphoinositide 4-kinases
PI4KB	Phosphatidylinositol-4-kinase-IIIβ
PKC	Protein kinase C
RAF	Rapidly Accelerated Fibrosarcoma
RhoA	Ras Homolog Family Member A
